# Use of antihypertensive drugs and risk of cutaneous melanoma: a nationwide nested case-control study

**DOI:** 10.1093/ije/dyac223

**Published:** 2022-11-22

**Authors:** Reza Ghiasvand, Leon A M Berge, Bettina K Andreassen, Jo S Stenehjem, Trond Heir, Øystein Karlstad, Asta Juzeniene, Inger K Larsen, Adele C Green, Marit B Veierød, Trude E Robsahm

**Affiliations:** Department of Research, Cancer Registry of Norway, Oslo, Norway; Oslo Centre for Biostatistics and Epidemiology, Oslo University Hospital, Oslo, Norway; Institute for Cancer Research, Oslo University Hospital, Oslo, Norway; Department of Research, Cancer Registry of Norway, Oslo, Norway; Institute for Cancer Research, Oslo University Hospital, Oslo, Norway; Oslo Centre for Biostatistics and Epidemiology, University of Oslo, Oslo, Norway; Department of Research, Cancer Registry of Norway, Oslo, Norway; Department of Research, Cancer Registry of Norway, Oslo, Norway; Institute of Clinical Medicine, University of Oslo, Oslo, Norway; Oslo Ischemia Study, Oslo University Hospital, Oslo, Norway; Department of Chronic Diseases and Ageing, Norwegian Institute of Public Health, Oslo, Norway; Department of Radiation Biology, Institute for Cancer Research, Oslo University Hospital, Oslo, Norway; Department of Registration, Cancer Registry of Norway, Oslo, Norway; Population Health Department, QIMR Berghofer Medical Research Institute, Brisbane, QLD, Australia; Cancer Research UK Manchester Institute and Faculty of Biology, Medicine and Health, Manchester Academic Health Sciences Centre, Manchester, UK; Oslo Centre for Biostatistics and Epidemiology, University of Oslo, Oslo, Norway; Department of Research, Cancer Registry of Norway, Oslo, Norway; Institute for Cancer Research, Oslo University Hospital, Oslo, Norway

**Keywords:** Antihypertensive drugs, melanoma, photosensitivity, ultraviolet radiation

## Abstract

**Background:**

Most antihypertensives can induce dermal photosensitivity, which may increase melanoma risk. However, corroborating evidence is limited. We examined the associations between use of antihypertensives and melanoma risk.

**Methods:**

A nationwide nested case-control study was conducted using data from the Cancer Registry of Norway, the National Registry and the Norwegian Prescription Database in 2004–15. Ten controls were randomly selected for each melanoma case, matched on sex and birth year. The study included 12 048 cases and 117 895 controls. We estimated rate ratios (RRs) with 95% confidence intervals (CIs). All analyses were adjusted for ambient ultraviolet radiation (UVR). We additionally performed active comparator analyses, and sensitivity analyses by only including new users, distinguishing between exclusive and mixed users, allowing for different latency periods, and subgroup analyses by melanoma subtype and clinical stage.

**Results:**

Compared with non-use, we observed a slightly increased melanoma risk in users of diuretics (RR 1.08, CI 1.01–1.15), calcium-channel blockers (RR 1.10, CI 1.04–1.18) and drugs affecting the renin-angiotensin system (RR 1.10, CI 1.04–1.16), but not for beta blockers (RR 0.97, CI 0.92–1.03). We found no heterogeneity of associations by melanoma subtype or clinical stage and no dose-response relationship between the cumulative defined daily doses (DDDs) and melanoma. No interaction was found between cumulative DDDs and ambient UVR.

**Conclusions:**

Weak associations, with lack of a dose-response relationship and lack of interactions with ambient UVR, in the DDD analysis in this nationwide study do not support a causal relationship between antihypertensives and melanoma risk.

Key Messages

- In this nationwide nested case-control study, we observed positive associations between melanoma development and use of diuretics, calcium-channel blockers and agents affecting the renin-angiotensin system, compared with non-use.
- We found no dose-response relationship between the cumulative dose of any antihypertensive drug and melanoma risk, or interaction between cumulative dose and ambient ultraviolet radiation (UVR).
- Weak associations and lack of a dose-response relationship in this study do not support a causal relationship between antihypertensive drugs and melanoma.

## Introduction

The incidence of cutaneous melanoma (hereafter, melanoma), the most lethal form of skin cancer, has increased markedly in older age groups in fair-skinned populations during the past decades.^1^ Excessive sun exposure is the foremost preventable risk factor and is responsible for the majority of melanomas.^2^ Melanoma development also depends on biological host^3^ and lifestyle factors,^4–9^ which may include the use of commonly prescribed drugs with photosensitizing properties. Drug-induced photosensitivity is an adverse dermatological reaction stemming from an interaction between ultraviolet radiation (UVR) and certain drugs, which may increase skin cancer risk.^10^

Hypertension is one of the most prevalent health conditions worldwide,^11^ and antihypertensive drugs are commonly prescribed for the reduction of hypertension-related morbidity and mortality.^12^^,^^13^ The majority of antihypertensive drugs, namely diuretics, beta blockers, calcium-channel blockers and drugs affecting the renin-angiotensin system (RAS), can induce photosensitivity.^14^^,^^15^ The photosensitizing potential of a drug is determined by its chemical structure's ability to absorb UVR and its dose.^16^ It is plausible that widespread use of drugs with photosensitizing potential may partly contribute to the increased melanoma rates in predominantly fair-skinned populations.^17^ However, corroborating evidence is limited.^18^

In recent years, an increasing number of studies have examined the association between antihypertensive drug use and melanoma. However, there is still a substantial knowledge gap concerning the potential increased risk of melanoma related to antihypertensive drugs.^17–21^ Epidemiological studies have reported an association between use of diuretics and increased melanoma occurrence,^17^^,^^20^^,^^21^ particularly for thiazide diuretics, a first-line treatment for hypertension. Evidence on the association between melanoma and other antihypertensive drugs is by contrast limited and conflicting, with some studies suggesting increased melanoma risk,^19^^,^^22^^,^^23^ whereas other studies suggest no association.^18^^,^^24^^,^^25^

To our knowledge, no nationwide epidemiological studies have investigated the associations between prescribed antihypertensive drugs with photosensitizing potential and melanoma, including analyses by histopathological subtypes, site and clinical stage at diagnosis, while taking residential ambient UVR exposure into account. Therefore, we aimed to investigate these associations employing population-based registries in Norway.

## Material and methods

Based on the principle of data minimization, we conducted a nested case-control study with a study population of ∼3.9 million adult residents (aged 18–85 years) in Norway between 2004 and 2015, registered in the Norwegian National Population Registry. We linked data from the Cancer Registry of Norway (CRN), the Norwegian Prescription Database (NorPD) and the Medical Birth Registry of Norway, combining them through the unique personal identification number assigned to all residents.^26^

### Cases and controls

The CRN has been recording all neoplasms diagnosed in Norway since 1953. Information from several independent sources (such as clinical and pathology reports and death certificates) ensures complete and high-quality data (>99% of melanomas diagnosed after 2000 were morphologically verified).^27^^,^^28^ Cancer diagnoses are recorded according to the International Classification of Diseases (ICD) 7th edition, ICD of oncology 2nd and 3rd edition, and converted to the ICD 10th revision. For this study, we included all first primary invasive melanoma diagnoses (C43, Supplementary Material, available as Supplementary data at *IJE* online) in Norway in 2007–15 in persons aged 18–85 years. Tumour site was categorized as head/neck, trunk, upper limb, lower limb,and other sites, including unspecified. Histological subtype was categorized as superficial spreading melanoma (SSM), nodular melanoma (NM) or other subtypes. Based on clinical and pathological information on metastasis, the CRN records clinical stage at diagnosis as local disease (no metastases), regional metastasis (regional lymph nodes, satellites and in-transit metastases), distant metastasis (non-regional lymph node and organ metastases), and unspecified stage.

**Figure 1:**
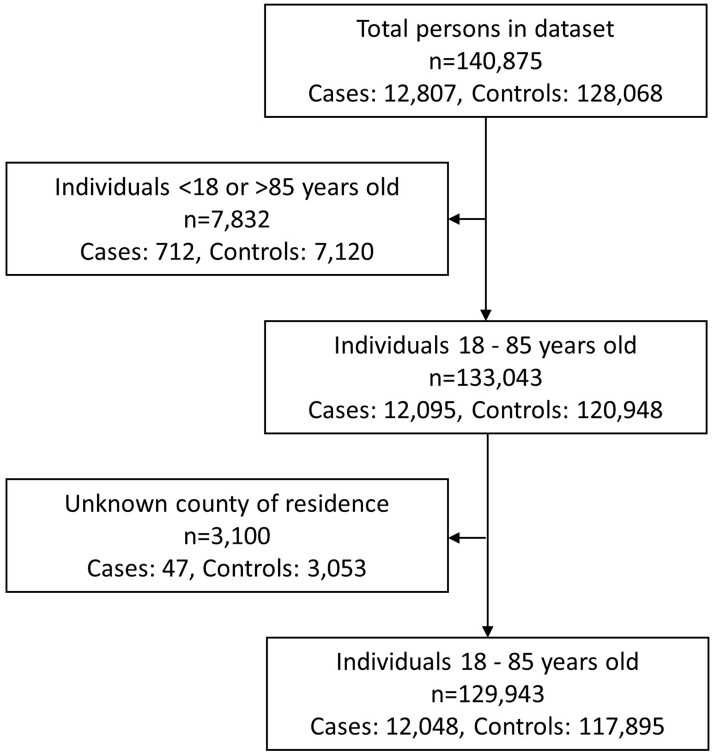
Flow diagram of the study sample

For each of the 12 807 melanoma cases, 10 controls were randomly selected (with replacement) from the National Population Registry, using risk-set sampling. Controls were matched on sex and birth year and had to be alive, residing in Norway and with no cancer history (except for basal cell carcinoma) at the index date (date of the melanoma diagnosis for the matched case). However, a cancer diagnosis afterwards was allowed. We excluded individuals aged <18 and >85 years (*n* = 7832) and those with unknown region of residence (*n* = 3100). The final study sample consisted of 129 943 individuals, including 12 048 cases and 117 895 controls (Figure 1).

### Prescribed drugs

The NorPD has recorded all prescription drugs dispensed from Norwegian pharmacies to patients outside hospitals and institutions since 1 January 2004. Prescriptions are classified according to the Anatomical Therapeutic Chemical (ATC) classification system version 2017.^29^^,^^30^ The date and information regarding the drug, the prescriber and the patient are recorded for each dispensing. For all prescriptions dispensed for cardiovascular disease (CVD) indications (included in the ATC classes C01-C10),^26^ we obtained dates of dispensing, the ATC code and the number of defined daily doses (DDD) for the period 2004–15. DDD was defined as the average maintenance dose per day for a drug used for its main indication in adults.^31^ For this study, we identified all prescribed antihypertensive drugs according to 2nd-level ATC class, including diuretics (C03), beta blockers (C07), calcium-channel blockers (C08) and RAS agents (C09).

The total number of filled prescriptions since 1 January 2004 (on separate or same dates) was summed for each individual and the antihypertensive drug use was defined as two or more filled prescriptions for each of the ATC classes. In addition, we summed thiazide diuretics (ATC: C03BB) that are specifically suggested to cause photosensitivity and increased risk of skin cancer.^32^ Filling only one prescription indicates limited use and was assumed not to affect cancer development; thus, the non-use category was defined as one or no prescription. To examine the potential dose-response associations, we calculated the individual cumulative dose for each antihypertensive class based on the total number of DDDs filled, and categorized it as non-user (DDD 0) or in tertiles of use.

To secure a prospective temporal relationship between exposure and disease, we collected drug use information at least 3 years before the melanoma diagnosis. Besides, we attempted to reduce the potential impact of reverse causality by disregarding prescriptions filled less than 1 year before the diagnosis/index dates. Additionally, since antihypertensive polytherapy is expected and we found correlations between the prescriptions of different drugs (Supplementary Table S1, available as Supplementary data at *IJE* online), users were categorized as mixed users if they used a combination of antihypertensive drugs and exclusive users if they exclusively used only one drug.

### Covariates

Norway has a distinct gradient in the ambient UVR dose, decreasing from southern to northern latitudes.^33^ Information on the region of residence was used as a proxy for ambient UVR exposure and was categorized as: highest (eastern and southern Norway), medium (western and central Norway), and lowest (northern Norway) levels of ambient UVR exposure.^9^

### Statistical analyses

To examine the association between antihypertensive drugs and melanoma risk, we applied conditional logistic regression, estimating rate ratios (RRs) with 95% confidence intervals (CIs). All the analyses were controlled for sex, year of birth and diagnosis/index date by design. RRs were additionally adjusted for residential ambient UVR and for the use of additional antihypertensive drugs other than the drug in focus. Statistical interactions between drug use and age, sex and ambient UVR were performed by adding interaction terms in the models and using a likelihood ratio test. When interaction was apparent, we conducted stratified analyses. Further, we tested whether exposure-disease associations differed by body site, histological subtype and clinical stage, using a contrast test (test for heterogeneity).^35^

Since non-user comparators might induce detection bias because of, for example, differential frequency of medical visits, and possible unmeasured confounding, we also used active comparator design,^34^ by: (i) comparing melanoma risk among the users of selected antihypertensive drugs with the users of other cardiovascular drugs (ATC codes C01–10); and (ii) with the users of alpha- and beta blockers (ATC codes C02AB, C02AC, C02CA, C07). Since prescription data were left-truncated (no information before 1 January 2004), we also performed sensitivity analyses by only including users with the first filled prescription after January 2004. Furthermore, due to the uncertain latency of melanoma development, we conducted sensitivity analyses allowing for a latency period of 2, 5 or 7 years. Even though we adjusted for several potential confounders, we cannot rule out uncontrolled confounding. Thus, generalized E-value (G-value) analysis was conducted to assess the robustness of the associations, as proposed by MacLehose *et al*.^35^ All statistical analyses were conducted using STATA (version 16).

## Results

### Characteristics of cases and controls

RAS agents were the most prescribed antihypertensive group, used by 26% of cases and 24% of controls (Table 1). The use of diuretics and calcium-channel blockers was slightly higher in cases than in controls (13.1% vs 12.0% and 25.5% vs 23.9%, respectively). The use of beta blockers, calcium-channel blockers and RAS agents was more common in men, and diuretics were slightly more common in women. The majority of drug users were ≥70 years of age (Table 1).

**Table 1:** Characteristics of cases (*n* = 12 048) and controls (*n* = 117 895) in the study sample

	Diuretics (ATC C03)^a^		Beta blockers (ATC C07)^a^		Calcium-channel blockers (ATC C08)^a^		Renin-angiotensin system agents (ATC C09)^a^		Complete study sample	
Characteristics	Cases	Controls	Cases	Controls	Cases	Controls	Cases	Controls	Cases	Controls
	*n* (%)	*n* (%)	*n* (%)	*n* (%)	*n* (%)	*n* (%)	*n* (%)	*n* (%)	*n* (%)	*n* (%)
Non-users	10 492 (87.1)	103 613 (87.9)	9792 (81.3)	97 547 (81.0)	10 465 (86.9)	103 695 (88.0)	8979 (74.5)	89 774 (76.1)	–	–
Users^b^	1556 (12.9)	14 282 (12.1)	2256 (18.7)	22 348 (19.0)	1583 (13.1)	14 200 (12.0)	3069 (25.5)	28 121 (23.9)	–	–
Use by cumulative dose^c^										
1st tertile	534 (4.4)	4839 (4.1)	541 (4.5)	4877 (4.2)	897 (7.4)	8263 (7.0)	1396 (11.6)	14 098 (12.0)	–	–
2nd tertile	542 (4.5)	4810 (4.1)	495 (4.1)	4933 (4.2)	860 (7.1)	7941 (6.7)	1546 (12.8)	13 967 (11.9)	–	–
3rd tertile	495 (4.1)	4867 (4.1)	454 (3.8)	4973 (4.2)	865 (7.2)	8110 (6.9)	1473 (12.2)	14 001 (11.9)	–	–
Use by sex										
Men	741 (12.4)	6602 (11.4)	1316 (22.1)	12 937 (22.3)	961 (16.1)	8135 (14.1)	1776 (39.8)	15 954 (27.6)	5957 (49.4)	57 897 (49.1)
Women	815 (13.4)	7680 (12.8)	940 (15.4)	9411 (15.7)	622 (10.2)	6065 (10.1)	1293 (21.2)	12 167 (20.3)	6091 (50.6)	59 998 (50.9)
Use by age										
Mean (SD)	70.0 (10.9)	70.4 (10.4)	69.8 (10.4)	69.7 (10.3)	70.2 (9.6)	70.4 (9.6)	68.6 (10.3)	68.8 (10.1)	59.5 (14.6)	59.6 (14.6)
<50 years	80 (2.5)	630 (2.1)	108 (3.4)	964 (3.2)	45 (1.4)	413 (1.4)	149 (4.7)	1201 (4.0)	3160 (26.2)	30 210 (25.6)
50–69 years	593 (10.6)	5468 (9.9)	896 (16.0)	9268 (16.9)	670 (12.0)	5801 (10.6)	1417 (25.4)	13 024 (23.7)	5587 (46.4)	54 983 (46.6)
≥70 years	883 (26.8)	8184 (25.0)	1252 (37.9)	12 116 (37.1)	868 (26.3)	7986 (24.4)	1503 (45.5)	13 896 (42.5)	3301 (27.4)	32 702 (27.7)
Use by region of ambient UVR^d^										
Lowest (Northern Norway)	98 (13.5)	1691 (14.3)	151 (20.9)	2712 (22.9)	93 (12.8)	1680 (14.2)	165 (22.8)	2997 (25.3)	9829 (81.6)	89 424 (75,9)
Medium (Central Norway)	188 (12.6)	1875 (11.3)	258 (17.3)	2951 (17.8)	180 (12.0)	1951 (11.7)	359 (24.0)	3570 (21.5)	1495 (12.4)	16 616 (14.1)
Highest (Southern Norway)	1270 (12.9)	10 716 (12.0)	1847 (18.8)	16 685 (18.7)	1310 (13.3)	10 569 (11.8)	2545 (25.9)	21 554 (24.1)	724 (6.0)	11 855 (10.0)

a According to the Anatomical Therapeutic Chemical (ATC) Classification maintained by the World Health Organization.

b Two or more prescriptions of the drug group.

c Defined daily dose (DDD). Total number of DDDs categorized into non-use (0), and tertiles of use, for diuretics as: 1–275, 276–1584, ≥1585; for beta blockers as: 1–360, 361–1272, ≥1273; for calcium-channel blockers as: 1–496, 497–2114, ≥2115; and for renin angiotensin system agents as: 1–1176, 1177–3190, ≥3191.

d UVR, ultraviolet radiation.

### Association between antihypertensive drugs and melanoma risk

**Figure 2:**
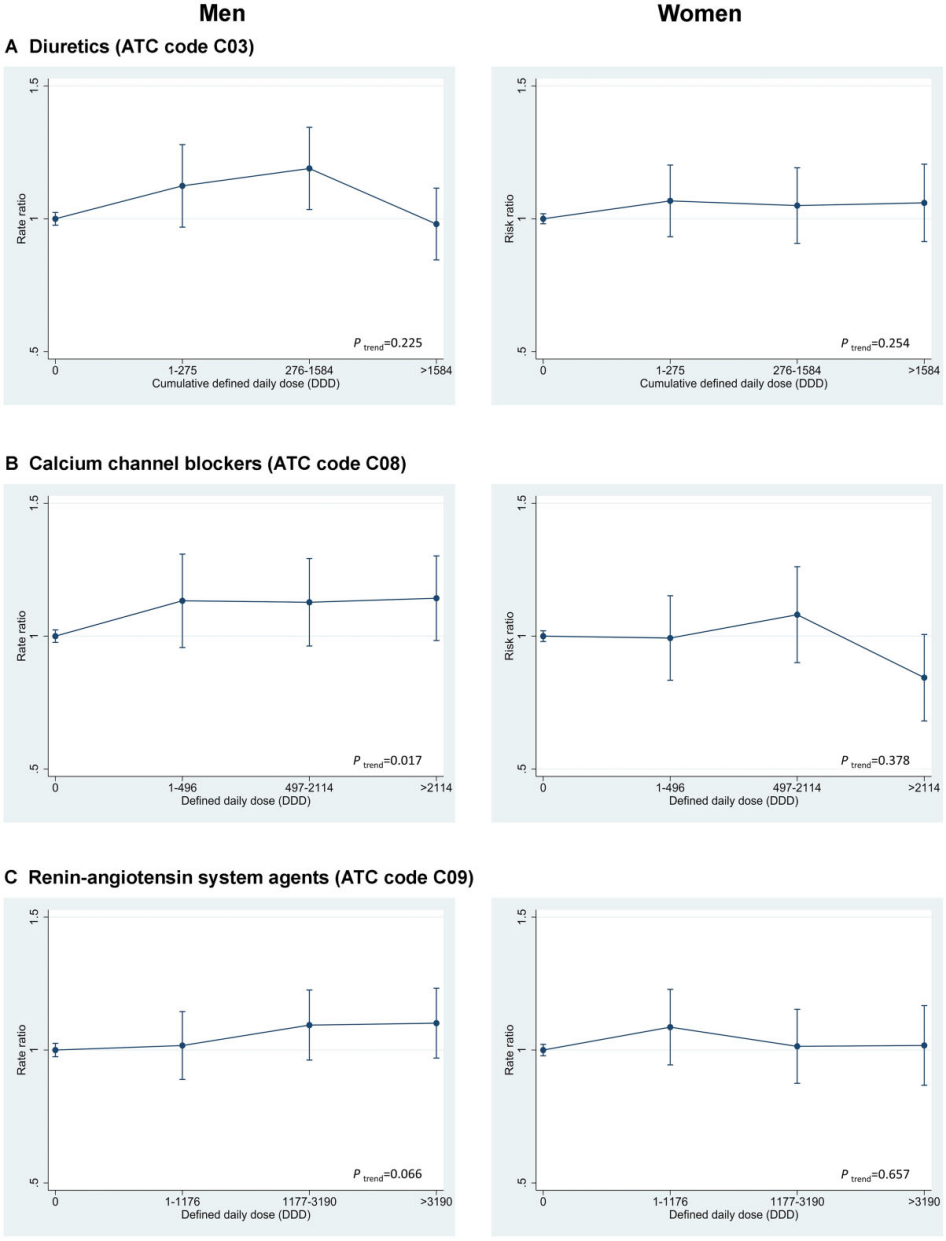
Dose-response patterns of diuretics, calcium-channel blockers and renin-angiotensin system agents, and risk of melanoma. ATC, Anatomical Therapeutic Chemical classification system version 2017

Compared with non-use, we found an elevated risk of melanoma in users of diuretics (RR 1.08, CI 1.01–1.15), calcium-channel blockers (RR 1.10, CI 1.04–1.18) and RAS agents (RR 1.10, CI 1.04–1.16) (Table 2). When analysed by sex, the increased risk associated with calcium-channel blockers was restricted to men (RR 1.18, CI 1.08–1.28; *P*_interaction_ = 0.006). RAS agents were associated with increased melanoma risk in regions with the highest (RR 1.09, CI 1.02–1.16) and medium ambient (RR 1.42, CI 1.15–1.75) UVR exposure and not the region with lowest ambient UVR exposure (RR 0.70, CI 0.49–1.01; *P*_interaction_ = 0.031). We found no dose-response associations between cumulative DDD of antihypertensives and melanoma risk. However in the analyses by sex, we found indications of a dose-response association between calcium-channel blockers and RAS agents in men (Figure 2).

**Table 2:** Rate ratios (RRs) with 95% confidence intervals (CIs) for melanoma risk in users of antihypertensive drugs vs non-users

Drug	Cases/controls	RR (95% CI)^a^
Diuretics (ATC C03)^b^		
Non-users	10 492/103 613	1.00
Users^c^	1556/14 282	1.08 (1.01–1.15)
Beta blockers (ATC C07)^b^		
Non-users	9792/95 547	1.00
Users^c^	2256/22 348	0.97 (0.92–1.03)
Calcium-channel blockers (ATC C08)^b^		
Non-users	10 465/103 695	1.00
Users^c^	1583/14 200	1.10 (1.04–1.18)
By sex		
Men	961/8135	1.18 (1.08–1.28)
Women	622/6065	1.01 (0.92–1.12)
*P* for interaction		*P *=* *0.006
Renin-angiotensin system agents (ATC C09)^b^		
Non-users	8979/89 774	1.00
Users^c^	3069/28 121	1.10 (1.04–1.16)
By region of ambient UVR^d^ exposure		
Lowest (Northern Norway)	165/2997	0.70 (0.49–1.01)
Medium (Central Norway)	359/3570	1.42 (1.15–1.75)
Highest (Southern Norway)	2545/21 554	1.09 (1.02–1.16)
*P* for interaction		*P *= 0.031

a Adjusted for region of ambient ultraviolet radiation exposure and all cardiovascular disease (CVD) medications (ATC code C).

b According to the Anatomical Therapeutic Chemical (ATC) Classification maintained by the World Health Organization.

c Two or more prescriptions of the drug group.

d UVR, ultraviolet radiation.

### Sensitivity analyses

The results of the active comparator analyses were comparable with the full case-control analysis for all antihypertensives, except for RAS agents, where the increased risk was found for men only (RR 1.09, CI 1.00–1.19) (Supplementary Tables S2 and S3, available as Supplementary data at *IJE* online). We found no interaction with age in the total sample and active comparator analyses.

In the analyses of mixed and exclusive use compared with non-use, increased melanoma risk was found in exclusive users of diuretics (RR 1.14, CI 1.01–1.28) and in mixed users of calcium-channel blockers (RR 1.12, CI 1.04–1.20) (Supplementary Table S4, available as Supplementary data at *IJE* online). For RAS agents, an increased risk was found for both mixed (RR 1.07, CI 1.00–1.15) and exclusive (RR 1.14, CI 1.05–1.23) use. In analyses by body site, the use of diuretics was associated with an increased melanoma risk on the trunk (RR 1.13, CI 1.03–1.24) and lower limb (RR 1.10, CI 0.96–1.26) but with no heterogeneity between sites (*P*_heterogeneity_ = 0.45; Table 3). The use of calcium-channel blockers was associated with an increased melanoma risk at all body sites except head/neck. RAS agents were associated with an increased melanoma risk on the upper limbs (RR 1.22, CI 1.05–1.41) and head/neck (RR 1.29, CI 1.11–1.49), but not the other sites (*P*_heterogeneity_=0.045; Table 3). For histological subtypes, we found associations between RAS agents and SSM (RR 1.14, CI 1.06–1.23) and between calcium-channel blockers and histological subtypes classified as other (RR 1.16, CI 1.03–1.30). However, we found no heterogeneity of association between antihypertensive drugs and melanoma subtypes (0.11≤*P*_heterogeneity_ ≤0.76). Additional analyses of thiazide diuretics showed no association with melanoma risk (Supplementary Table S5, available as Supplementary data at *IJE* online). The results of the analysis when we only included users with the first filled prescription after January 2004 were similar to the main analysis (Supplementary Table S6, available as Supplementary data at *IJE* online). Moreover, allowing for a latency period of 2, 5 or 7 years did not affect the estimates (Supplementary Table S7, available as Supplementary data at *IJE* online). We used the RR for calcium-channel blockers and melanoma risk in Table 2 (the largest observed RR in overall analysis: 1.10) for G-value calculations. Assuming different prevalence of an uncontrolled confounder among users and non-users of the drug, we estimated that an uncontrolled confounder associated with both drug use and melanoma risk by an RR of between 1.43-fold and 2.69-fold each would explain the observed association between calcium-channel blockers and melanoma risk, if no causal association existed (Supplementary Table S8, available as Supplementary data at *IJE* online).

**Table 3:** Rate ratios (RRs) with 95% confidence intervals (CIs) for melanoma risk in users of diuretics, calcium-channel blockers and renin-angiotensin system agents vs non-users, by anatomical site, subtype and metastasis

	Diuretics (ATC C03)^a^		Calcium-channel blockers (ATC C08)^a^		Renin-angiotensin system agents (ATC C09)^a^	
	Cases/controls	RR (95% CI)^b^	Cases/controls	RR (95% CI)^b^	Cases/controls	RR (95% CI)^b^
**By anatomical site**						
Trunk						
Non-users	4932/48 657	1.00	4881/48 268	1.00	4204/41 777	1.00
Users^c^	688/6048	1.13 (1.03–1.24)	739/6437	1.13 (1.03–1.24)	1416/12 928	1.06 (0.98–1.15)
Lower limb						
Non-users	2524/24 804	1.00	2571/25 195	1.00	2281/22 332	1.00
Users^c^	337/3120	1.10 (0.96–1.26)	290/2729	1.08 (0.93–1.25)	580/5592	1.04 (0.92–1.17)
Upper limb						
Non-users	1330/13 048	1.00	1326/13 164	1.00	1118/11 360	1.00
Users^c^	225/2104	1.01 (0.86–1.20)	229/1988	1.12 (0.95–1.33)	437/3792	1.22 (1.05–1.41)
Head/neck						
Non-users	1097/10 752	1.00	1098/10 721	1.00	870/8919	1.00
Users^c^	215/2062	0.99 (0.84–1.18)	214/2093	0.96 (0.81–1.14)	442/3895	1.29 (1.11–1.49)
*P* for heterogeneity		0.45		0.43		0.045
**By subtype**						
Superficial spreading melanoma						
Non-users	5878/57 994	1.00	5878/58 088	1.00	5082/50 989	1.00
Users^c^	751/7012	1.05 (0.96–1.15)	751/6918	1.06 (0.96–1.16)	1547/14 017	1.14 (1.06–1.23)
Nodular melanoma						
Non-users	1755/17 372	1.00	1736/17 338	1.00	1463/14 708	1.00
Users^c^	316/2878	1.07 (0.93–1.23)	335/2912	1.14 (0.99–1.32)	608/5542	1.09 (0.97–1.24)
Other histological types						
Non-users	2859/28 287	1.00	2851/28 322	1.00	2434/24 166	1.00
Users^c^	489/4352	1.11 (0.99–1.25)	497/4317	1.16 (1.03–1.30)	914/8473	1.05 (0.95–1.16)
*P* for heterogeneity		0.76		0.11		0.45
**By stage of disease**						
Local disease						
Non-users	8535/84 298	1.00	8535/84 420	1.00	7327/73 209	1.00
Users^c^	1248/11 499	1.07 (1.00–1.15)	1248/11 377	1.08 (1.01–1.16)	2456/22 588	1.10 (1.03–1.17)
Regional metastases						
Non-users	473/4725	1.00	466/4704	1.00	380/3911	1.00
Users^c^	94/828	1.07 (0.83–1.40)	101/849	1.13 (0.87–1.47)	187/1642	1.16 (0.92–1.46)
Distant metastases						
Non-users	548/5343	1.00	535/5318	1.00	442/4466	1.00
Users^c^	84/843	0.96 (0.73–1.25)	97/868	1.11 (0.86–1.44)	190/1720	1.15 (0.93–1.44)
Unspecified						
Non-users	936/9287	1.00	929/9306	1.00	830/8277	1.00
Users^c^	130/1072	1.23 (0.99–1.53)	137/1053	1.33 (1.07–1.66)	236/2082	1.06 (0.88–1.28)

a According to the Anatomical Therapeutic Chemical (ATC) Classification maintained by the World Health Organization.

b Adjusted for region of ambient ultraviolet radiation exposure and all cardiovascular disease (CVD) medications (ATC code C).

c Two or more prescriptions of the drug group.

## Discussion

In this nationwide nested case-control study, we observed a weak positive association between melanoma risk and use of diuretics, calcium-channel blockers and RAS agents, compared with non-use. These associations tended to be confined to men. The use of RAS agents was associated with increased melanoma risk in regions with highest and medium, but not lowest, ambient UVR. However, we found no dose-response relationship between the cumulative dose of any antihypertensive drug and melanoma risk, nor interaction between cumulative dose and ambient UVR.

The weak association found between diuretics and melanoma risk is in line with a recent meta-analysis.^19^ However, the lack of dose-response associations in our study does not support a causal association with diuretic use, consistent with a review of contemporary literature,^18^ and with a cohort study that reported no overall or dose-response association between hydrochlorothiazide and melanoma risk.^36^ Among antihypertensive drugs, diuretics may have the greatest potential for triggering photosensitizing reactions, causing DNA damage and skin inflammation,^37^ and studies have examined diuretic use in relation to melanoma risk.^36–39^ The use of selected thiazide diuretics may increase the risk,^38^^,^^39^ particularly for nodular melanoma.^21^ However, we found no association between thiazide diuretics and melanoma risk, either in the overall analysis or by histological subtype.

Beta blockers also have photosensitizing properties,^40^ but few studies have examined the association between use of beta blockers and melanoma, with the results being inconclusive. Our finding of no association with melanoma corresponds with findings from a large cohort study.^24^ A case-control study, however, found 15% increased melanoma risk among users of beta blockers.^22^ A meta-analysis also reported increased melanoma risk associated with beta blockers.^19^ However, the estimate was based on three studies. On the other hand, preclinical studies have demonstrated anti-cancer effects in melanoma cells from non-selective beta blockers such as propranolol.^21^^,^^41^ Thus, beta blockers' potential beneficial effect may cancel out their harmful photosensitizing effect.

The literature on calcium-channel blockers and melanoma risk is also inconclusive.^19^ A meta-analysis of observational studies suggested a weak association between calcium-channel blockers and melanoma risk.^23^ However, a randomized controlled trial reported no association.^25^ In our study, calcium-channel blockers were associated with an increased risk of melanoma in men but not women; however, no dose-response association was found.

The photosensitizing potential of RAS agents is well documented,^42^ although few studies have examined its association with melanoma risk, with inconclusive results.^19^^,^^23^ Compared with non-use, RAS agent use was associated with increased melanoma risk particularly for upper limb and head and neck melanomas, for men and among those living in regions with medium and highest ambient UVR. Together, this suggests that RAS agents might increase melanoma risk through the chronic sun exposure pathway.^43^ On the other hand, the lack of a dose-response relation and the interaction with ambient UVR exposure in the DDD analysis make any causal interpretation of the results questionable.

Major strengths of this study include high-quality data regarding all first primary melanoma diagnoses for adults residing in Norway and comprehensive information regarding prediagnostic drug use. The NorPD records information on drugs dispensed from pharmacies to patients, limiting the potential impact of primary non-adherence. We attempted to control for reverse causation bias by excluding prescriptions ≤1 year before the diagnosis/index date.

The study has weaknesses to be considered. Uncontrolled confounding remains a possibility due to the observational nature of this study. We estimated that an uncontrolled confounder or a set of uncontrolled confounders, associated with both drug use and risk of melanoma by an RR between 1.43 and 2.69, could completely explain the largest observed association in the overall analysis (RR = 1.10). Imperfect measurement of confounders, such as using residential UVR exposure as a proxy for personal UVR exposure which, apart from causing melanoma, interacts with photosensitizing drugs, and potential confounders not included in the analysis (such as body mass index) may thus explain observed associations. Indications for drug use may also be a source of confounding, as the melanoma risk might be indirectly modified by the illness severity, through UVR exposure and by diagnostic intensity. We attempted to account for these issues by active comparator analyses and by separating exclusive and mixed users. We cannot disentangle whether intra-regional differences concerning prescription practices and access to health care have influenced the results, but adjustment for the use of other CVD drugs could act as a proxy in this regard. We tested for interaction between drug use and residential ambient UVR, and when absent, we adjusted for the potential confounding effect of UVR. Nevertheless, the lack of interaction between photosensitizing drugs and UVR in our study might indicate that residential UVR is not a perfect proxy for personal UVR exposure. Additionally, small samples in sensitivity analyses might have provided insufficient power to detect associations. The interval between drug use and melanoma is uncertain and may lead to exposure misclassification. However, latency periods of 2, 5 or 7 years did not affect the estimates, which further supports a non-causal association between antihypertensive drug use and melanoma. This study may also suffer from a short follow-up from the start of drug use, or time-window bias. We did not have information about drug use before 2004, which may have introduced drug use misclassification. However, the results of the sensitivity analysis, restricted to users with the first filled prescription after January 2004, were similar to those of the main analysis. Furthermore, hypertension polytherapy is common, and the combinations of drugs used may change over time. Approximately 3% of controls were excluded from the analysis due to missing residential information, which was sourced by NorDP. Controls who lacked this information did not receive any prescription drugs. Thus, this exclusion may have introduced a certain degree of selection bias. However, comparable results from the main analyses and active comparator analyses suggest otherwise.

To our knowledge, this is the first nationwide register-based study examining the association between antihypertensive drugs with photosensitizing properties and risk of melanoma, by histopathological subtypes, site and clinical stage. Our findings suggest associations between diuretics, calcium-channel blockers and RAS agents with melanoma risk, mainly among men. However, small effect sizes with lack of a dose-response association, and lack of interaction with ambient UVR exposure in the DDD analysis, do not support a causal association.

## Ethics approval

The study is approved by the Norwegian Data Protection Authority and the Regional Committee for Medical and Health Research Ethics (no. 2017/1246). The study is also approved by the national registries contributing with data: CRN, the National Registry, NorPD and the Medical Birth Registry. The linkage key for the 11-digit PINs was stored and governed by a third party unavailable to the research team. All data management and analyses were conducted on data with no individual person identified. This case-control study used only data from nationwide population-based registers, and thus did not include a recruitment process for patients, who were involved in neither the design nor the conduct of the study. Thus, the research question and outcome measures were not informed by any specific patient priorities, experiences or preferences. Rather, their formulation was based upon our own priorities for patient benefit and result interpretation. All results are distributed on a group level, without any possibilities for individual identification.

## Supplementary Material

dyac223_Supplementary_Data

## Data Availability

Requests for data sharing should be directed to the corresponding author. This project uses third-party data derived from state government registries, which are ultimately governed by their ethics committees and data custodians. Thus, any requests to share these data will be subject to formal approval from each data source used in the project, as well as from the Data Protection Authority and Regional Committees for Medical and Health Research Ethics, according to the General Data Protection Regulation.
