# Supplementary material for: Use of antihypertensive drugs and risk of cutaneous melanoma: a nationwide nested case-control study
Source: Int J Epidemiol. 2022 Nov 22;52(3):887–98. doi: 10.1093/ije/dyac223 (PMC10244056; doi:10.1093/ije/dyac223)
Supplement: dyac223_Supplementary_Data [file dyac223_supplementary_data.docx]

| **Supplementary Table S1.** Correlation^a^ between the number of prescriptions of different drug groups | | | |
| --- | --- | --- | --- |
|  | Diuretics  (ATC C03) | Beta-blockers  (ATC C07) | Calcium channel blockers (ATC C08) |
| Beta-blockers (ATC C07) | 0.41 |  |  |
| Calcium channel blockers (ATC C08) | 0.36 | 0.40 |  |
| Renin-angiotensin system agents (ATC C09) | 0.44 | 0.43 | 0.55 |
| ^a^ Pearson correlation coefficient. | | | |

**ICD-10 codes:**

| **Site** | **ICD codes** |
| --- | --- |
| Cutaneous melanoma | C43 |
| Head/neck | C43.0 – C43.4 |
| Upper limb | C43.6 |
| Trunk | C43.5 |
| Lower limb | C43.7 |
| Other sites including unspecified | C43.8-C43.9 |

| Supplementary Table S2. Rate ratios (RRs) with 95% confidence intervals (CIs) for melanoma risk in users of antihypertensive drug groups from the active comparator analysis confined to users of cardiovascular disease medications | | | | | | | |
| --- | --- | --- | --- | --- | --- | --- | --- |
|  | **Overall** | | **Men** | | **Women** | | **P value^c^** |
|  | **Cases/controls^a^** | **RR(95% CI)^b^** | **Cases/controls^a^** | **RR(95% CI)^b^** | **Cases/controls^a^** | **RR(95% CI)^b^** |  |
| Diuretics (ATC C03) |  |  |  |  |  |  |  |
| Non-users | 3,621/35,126 | 1.00 | 2,054/19,985 | 1.00 | 1,567/15,141 | 1.00 |  |
| Users | 1,556/14,282 | 1.07 (1.00 – 1.15) | 741/6,602 | 1.10 (1.01 – 1.21) | 815/7,680 | 1.03 (0.93 – 1.14) | 0.28 |
| Beta-blockers (ATC C07) |  |  |  |  |  |  |  |
| Non-users | 2,921/27,060 | 1.00 | 1,479/13,650 | 1.00 | 1,442/13,410 | 1.00 |  |
| Users | 2,256/22,348 | 0.96 (0.90 – 1.02) | 1,316/12,937 | 0.94 (0.86 – 1.03) | 940/9,411 | 0.98 (0.89 – 1.07) | 0.86 |
| Calcium channel blockers (ATC C08) |  |  |  |  |  |  |  |
| Nonusers | 3,594/35,208 | 1.00 | 1,834/18,452 | 1.00 | 1,760/16,756 | 100 |  |
| Users | 1,588/14,200 | 1.10 (1.02 – 1.17) | 961/8,135 | 1.19 (1.09 – 1.30) | 622/6,065 | 0.98 (0.88 – 1.09) | 0.002 |
| Renin-angiotensin system agents (ATC C09) |  |  |  |  |  |  |  |
| Non-users | 2,108/21,287 | 1.00 | 1,019/10,633 | 1.00 | 1,089/10,654 | 1.00 |  |
| Users | 3,069/28,121 | 1.05 (0.98 – 1.12) | 1,776/15,954 | 1.09 (1.00 – 1.19) | 1,293/12,167 | 1.00 (0.91 – 1.10) | 0.081 |
| ^a^ Study sample was confined to users of cardiovascular disease medications (ATC code C); cases= 5,177 and controls=49,408.  ^b^ Adjusted for region of ambient ultraviolet radiation exposure and all cardiovascular disease medications.  ^c^ P for interaction. | | | | | | | |

| Supplementary Table S3. Rate ratios (RRs) with 95% confidence intervals (CIs) for melanoma risk in users of antihypertensive drug groups from the active comparator analysis confined to users of alpha and beta-blocker users (ATC codes C02AB, C02AC, C02CA, C07) | | | | | | | |
| --- | --- | --- | --- | --- | --- | --- | --- |
|  | **Overall** | | **Men** | | **Women** | | **P value^c^** |
|  | **Cases/controls^a^** | **RR(95% CI)^b^** | **Cases/controls^a^** | **RR(95% CI)^b^** | **Cases/controls^a^** | **RR(95% CI)^b^** |  |
| Diuretics (ATC C03) |  |  |  |  |  |  |  |
| Non-users | 1,510/15,488 | 1.00 | 916/9,489 | 1.00 | 1,567/15,141 | 1.00 |  |
| Users | 817/7,571 | 1.11 (1.01 – 1.21) | 455/4,018 | 1.15 (1.03 – 1.31) | 815/7,680 | 1.02 (0.86 – 1.22) | 0.13 |
| Beta-blockers (ATC C07) |  |  |  |  |  |  |  |
| Non-users | 71/711 | 1.00 | 55/570 | 1.00 | 1,442/13,410 | 1.00 |  |
| Users | 2,256/22,348 | 1.11 (0.77 – 1.61) | 1,316/12,937 | 1.12 (0.76 – 1.70) | 940/9,411 | 1.00 (0.45 – 2.22) | 0.62 |
| Calcium channel blockers (ATC C08) |  |  |  |  |  |  |  |
| Nonusers | 1,489/15,147 | 1.00 | 835/8,753 | 1.00 | 1,760/16,756 | 100 |  |
| Users | 838/7,912 | 1.06 (0.95 – 1.18) | 536/4,754 | 1.12 (0.97 – 1.28) | 622/6,065 | 0.97 (0.81 – 1.16) | 0.12 |
| Renin-angiotensin system agents (ATC C09) |  |  |  |  |  |  |  |
| Non-users | 890/9,207 | 1.00 | 488/5,127 | 1.00 | 1,089/10,654 | 1.00 |  |
| Users | 1,437/13,852 | 1.07 (0.96 – 1.20) | 883/8,380 | 1.05 (0.91 – 1.21) | 1,293/12,167 | 1.13 (0.94 – 1.34) | 0.87 |
| ^a^ Study sample: cases= 2,327 and controls=23,059.  ^b^ Adjusted for region of ambient ultraviolet radiation exposure and all cardiovascular disease medications.  ^c^ P for interaction. | | | | | | | |

| Supplementary Table S4. Rate ratios (RRs) with 95% confidence intervals (CIs) for use of antihypertensive drugs and melanoma risk | | |
| --- | --- | --- |
|  | **Cases/controls^a^** | **RR(95% CI)^b^** |
| Diuretics (C03) |  |  |
| Non-users | 10,492/103,613 | 1.00 |
| Mixed users^c^ | 1,251/11,517 | 1.06 (0.98 – 1.14) |
| Excusive users | 305/2,765 | 1.14 (1.01 – 1.28) |
| P for heterogeneity^d^ |  | 0.32 |
| Beta-blockers (C07) |  |  |
| Non-users | 9,792/95,547 | 1.00 |
| Mixed users^c^ | 1,694/16,606 | 0.93 (0.87 – 1.00) |
| Excusive users | 562/5,742 | 1.06 (0.96 – 1.16) |
| P for heterogeneity^d^ |  | 0.033 |
| Calcium channel blockers (C08) |  |  |
| Non-users | 10,465/103,695 | 1.00 |
| Mixed users^c^ | 1,418/12,540 | 1.12 (1.04 – 1.20) |
| Excusive users | 165/1,660 | 1.04 (0.89 – 1.23) |
| P for heterogeneity^d^ |  | 0.45 |
| Renin-angiotensin system agents (C09) |  |  |
| Non-users | 8,979/89,774 | 1.00 |
| Mixed users^c^ | 2,187/20,139 | 1.07 (1.00 – 1.15) |
| Excusive users | 882/7,982 | 1.14 (1.05 – 1.23) |
| P for heterogeneity^d^ |  | 0.24 |
| ^a^Study sample was confined to users of cardiovascular disease (CVD) medications (ATC code C); cases= 5,177 and controls=49,408.  ^b^Adjusted for region of ambient ultraviolet radiation exposure and all CVD medications.  ^c^Users who were also prescribed one or more other types of antihypertensive medication.  ^d^Test for heterogeneity between mixed and exclusive users. | | |

| **Supplementary Table S5.** Rate ratios (RRs) with 95% confidence intervals (CIs) for melanoma risk in users of thiazide diuretics | | | |
| --- | --- | --- | --- |
|  | **Cases/controls** | **RR(95% CI)^a^** | **RR(95% CI)^b^** |
| **Non-users** | 11,536/113,031 | 1.00 | 1.00 |
| **Users (≥2 prescriptions)** | 512/4,864 | 1.03 (0.94 – 1.13) | 1.01 (0.91 – 1.12) |
| **By sex** |  |  |  |
| Men | 241/2,245 | 1.03 (0.90 – 1.18) | 1.00 (0.86 – 1.16) |
| Women | 271/2,619 | 1.06 (0.89 – 1.11) | 1.02 (0.89 – 1.18) |
| **By anatomic site** |  |  |  |
| Trunk | 229/5,147 | 1.05 (0.91 – 1.20) | 1.02 (0.87 – 1.18) |
| Lower limb | 114/5,262 | 1.07 (0.88 – 1.21) | 1.09 (0.87 – 1.36) |
| Upper limb | 71/5,305 | 0.98 (0.77 – 1.24) | 0.98 (0.74 – 1.29) |
| Head/neck | 68/5,308 | 1.07 (0.86 – 1.34) | 0.94 (0.71 – 1.24) |
| **By subtype** |  |  |  |
| Superficial spreading melanoma | 262/5,114 | 1.05 (0.93 – 1.20) | 1.02 (0.88 – 1.18) |
| Nodular melanoma | 91/5,285 | 0.90 (0.73 – 1.10) | 0.89 (0.70 – 1.13) |
| Other types | 159/5,217 | 1.09 (0.92 – 1.30) | 1.08 (0.90 – 1.30) |
| **By stage of disease** |  |  |  |
| Local disease | 410/4,966 | 1.00 (0.91 – 1.11) | 0.97 (0.87 – 1.09) |
| Regional metastasis | 29/5,347 | 0.95 (0.66 – 1.37) | 0.93 (0.61 – 1.41) |
| Distant metastasis | 30/5,346 | 1.25 (0.86 – 1.83) | 1.44 (0.94 – 2.22) |
| Unspecified | 43/5,333 | 1.52 (1.09 – 2.12) | 1.35 (0.93 – 1.96) |
| ^a^ Adjusted for region of ambient ultraviolet radiation exposure and all antihypertensive drug groups.  ^b^ Active comparator analysis with study sample confined to users of cardiovascular disease (CVD) medications (ATC code C); cases= 5,177 and controls=49,408. | | | |

| **Supplementary Table S6.** Rate ratios (RRs) with 95% confidence intervals (CIs) for melanoma risk in users of antihypertensive drugs with the first filled prescription after January, 2004, versus non-users | | | | |
| --- | --- | --- | --- | --- |
|  | **Cases/controls** | **RR (95% CI)^a^** | **Cases/controls^d^** | **RR (95% CI)^a,d^** |
| **Diuretics (ATC C03)^b^** |  |  |  |  |
| Non-users | 10,492/103,613 | 1.00 | 3,312/32,299 | 1.00 |
| Users^c^ | 1,485/13,536 | 1.08 (1.02 – 1.16) | 1,402/12,794 | 1.08 (1.01 – 1.17) |
| **Beta-blockers (ATC C07)^b^** |  |  |  |  |
| Non-users | 9,792/95,547 | 1.00 | 2,670/24,779 | 1.00 |
| Users^c^ | 2,128/20,494 | 0.98 (0.92 – 1.04) | 2,044/20,314 | 0.96 (0.90 – 1.03) |
| **Calcium channel blockers (ATC C08)^b^** |  |  |  |  |
| Non-users | 10,465/103,695 | 1.00 | 3,347/32,847 | 1.00 |
| Users^c^ | 1,458/13,079 | 1.11 (1.04 – 1.19) | 1,367/12,246 | 1.10 (1.02 – 1.18) |
| **Renin-angiotensin system agents (ATC C09)^b^** |  |  |  |  |
| Non-users | 8,979/89,774 | 1.00 | 2,007/20,313 | 1.00 |
| Users^c^ | 2,780/25,418 | 1.10 (1.04 – 1.16) | 2,707/24,780 | 1.06 (0.99 – 1.13) |
| ^a^Adjusted for region of ambient ultraviolet radiation exposure and all cardiovascular disease (CVD) medications (ATC code C).  ^b^According to the Anatomical Therapeutic Chemical (ATC) Classification maintained by the World Health Organization.  ^c^ ≥2 prescriptions of the drug group.  ^d^Active comparator analysis confined to users of cardiovascular disease medications (ATC code C). Study sample: cases= 4,712 and controls=45,093. | | | | |

| **Supplementary Table S7.** Rate ratios (RRs) with 95% confidence intervals (CIs) for melanoma risk in users of antihypertensive drugs versus non-users with the application of a latency period of 2, 5, or 7 years between drug use and diagnosis/index date. | | | | | | |
| --- | --- | --- | --- | --- | --- | --- |
|  | **Cases/controls^b^** | **RR (95% CI)^a,b^** | **Cases/controls^c^** | **RR (95% CI)^a,c^** | **Cases/controls^d^** | **RR (95% CI)^a,d^** |
| **Diuretics (ATC C03)^e^** |  |  |  |  |  |  |
| Non-users | 10,646/105,051 | 1.00 | 11,409/112,158 | 1.00 | 11,554/113,731 | 1.00 |
| Users^f^ | 1,402/12,844 | 1.07 (1.01 – 1.15) | 639/5,737 | 1.09 (1.00 – 1.20) | 494/4,164 | 1.11 (1.02 – 1.31) |
| **Beta-blockers (ATC C07)^e^** |  |  |  |  |  |  |
| Non-users | 9,960/97,142 | 1.00 | 10,953/107,113 | 1.00 | 11,181/109,462 | 1.00 |
| Users^f^ | 2,088/20,753 | 0.96 (0.90 – 1.01) | 1,095/10,782 | 0.97 (0.89 – 1.05) | 867/8,433 | 0.99 (0.91 – 1.07) |
| **Calcium channel blockers (ATC C08)^e^** |  |  |  |  |  |  |
| Non-users | 10,613/104,960 | 1.00 | 11,342/111,512 | 1.00 | 11,511/113,079 | 1.00 |
| Users^f^ | 1,435/12,935 | 1.08 (1.01 – 1.15) | 706/6,383 | 1.07 (0.98 – 1.18) | 537/4,816 | 1.08 (0.99 – 1.19) |
| **Renin-angiotensin system agents (ATC C09)^e^** |  |  |  |  |  |  |
| Non-users | 9,213/92,018 | 1.00 | 10,554/104,485 | 1.00 | 10,874/107,394 | 1.00 |
| Users^f^ | 2,835/25,877 | 1.11 (1.05 – 1.17) | 1,494/13,412 | 1.11 (1.04 – 1.19) | 1,174/10,501 | 1.11 (1.03 – 1.20) |
| ^a^Adjusted for region of ambient ultraviolet radiation exposure and all cardiovascular disease (CVD) medications (ATC code C).  ^b^Prescriptions filled during the 2-year period prior to the diagnosis/index date were disregarded.  ^c^Prescriptions filled during the 5-year period prior to the diagnosis/index date were disregarded.  ^d^Prescriptions filled during the 7-year period prior to the diagnosis/index date were disregarded.  ^e^According to the Anatomical Therapeutic Chemical (ATC) Classification maintained by the World Health Organization.  ^f^ ≥2 prescriptions of the drug group. | | | | | | |

| Supplementary Table S8. The magnitude of risk ratio (RR) for the association between an uncontrolled confounder (or a set of uncontrolled confounders) with both drug use and melanoma required to reduce the observed association between drug use and melanoma to the null, generalized E-value (G-value), by prevalence of the uncontrolled confounder among users and nonusers. | | | | |
| --- | --- | --- | --- | --- |
|  |  | **RR=1.10^2^** | |  |
| P_1_^1^ |  | **G-value** | **P_0_^3^** |  |
| 0.1 |  | 2.69 | 0.04 |  |
| 0.2 |  | 2.07 | 0.10 |  |
| 0.3 |  | 1.83 | 0.16 |  |
| 0.4 |  | 1.70 | 0.23 |  |
| 0.5 |  | 1.62 | 0.31 |  |
| 0.6 |  | 1.56 | 0.38 |  |
| 0.7 |  | 1.52 | 0.46 |  |
| 0.8 |  | 1.48 | 0.54 |  |
| 0.9 |  | 1.46 | 0.62 |  |
| 1.0 |  | 1.43 | 0.70 |  |
| ^1^ P_1_, true prevalence of the uncontrolled confounder among users.  ^2^ RR for the association between calcium channel blockers and melanoma risk, the largest RR in Table 2.  ^3^ P_0_, implied prevalence of the uncontrolled confounder among nonusers. | | | | |
